# Serological responses triggered by different SARS-CoV-2 vaccines against SARS-CoV-2 variants in Taiwan

**DOI:** 10.3389/fimmu.2022.1023943

**Published:** 2022-11-15

**Authors:** Chiao-Hsuan Chao, Dayna Cheng, Sheng-Wen Huang, Yung-Chun Chuang, Trai-Ming Yeh, Jen-Ren Wang

**Affiliations:** ^1^ Department of Medical Laboratory Science and Biotechnology, College of Medicine, National Cheng Kung University, Tainan, Taiwan; ^2^ Institute of Basic Medical Sciences, College of Medicine, National Cheng Kung University, Tainan, Taiwan; ^3^ National Mosquito-Borne Diseases Control Research Center, National Health Research Institutes, Tainan, Taiwan; ^4^ Leadgene Biomedical, Inc., Tainan, Taiwan; ^5^ Center of Infectious Disease and Signaling Research, National Cheng Kung University, Tainan, Taiwan; ^6^ National Institute of Infectious Diseases and Vaccinology, National Health Research Institutes, Tainan, Taiwan

**Keywords:** COVID-19, vaccine, binding antibodies, neutralizing antibodies, anti-ACE2 antibodies

## Abstract

Broadly neutralizing ability is critical for developing the next-generation SARS-CoV-2 vaccine. We collected sera samples between December 2021-January 2022 from 113 Taiwan naïve participants after their second dose of homologous vaccine (AZD1222, mRNA-1273, BNT162-b2, and MVC-COV1901) and compared the differences in serological responses of various SARS-CoV-2 vaccines. Compared to AZD1222, the two mRNA vaccines could elicit a higher level of anti-S1-RBD binding antibodies with higher broadly neutralizing ability evaluated using pseudoviruses of various SARS-CoV-2 lineages. The antigenic maps produced from the neutralization data implied that Omicron represents very different antigenic characteristics from the ancestral lineage. These results suggested that constantly administering the vaccine with ancestral Wuhan spike is insufficient for the Omicron outbreak. In addition, we found that anti-ACE2 autoantibodies were significantly increased in all four vaccinated groups compared to the unvaccinated pre-pandemic group, which needed to be investigated in the future.

## Introduction

COVID-19 pandemic occurred at the end of 2019 and has caused 624 million infections and 6.5 million deaths as of 25 October 2022, according to the statistics of World Health Organization (WHO) (1). In addition to the serious health problems, the COVID-19 pandemic has caused a negative impact on the global economy (2). Scientists worldwide were dedicated to investigating the SARS-CoV-2-related research, especially in vaccines and antiviral drug developments to combat coronavirus. To date, there are eleven COVID-19 vaccines based on different platforms that have been granted emergency use listing (EUL) by the World Health Organization (WHO) (https://covid19.trackvaccines.org/agency/who/). The platforms of these granted vaccines include inactivated virus (Covaxin from Bharat Biotech, Covilo from Sinopharm (Beijing), and CoronaVac from Sinovac), non-replicating viral vector (Convidcia from CanSino, Ad26.COV2.S from Janssen, Vaxzevria (AZD1222, ChAdOx1 nCoV-19) from Oxford/AstraZeneca, and Covishield (Oxford/AstraZeneca formulation) from Serum Institute of India), RNA (Spikevax (mRNA-1273) from Moderna and Comirnaty (BNT162-b2) from Pfizer/BioNTech), and protein subunit (Nuvaxovid (NVX-CoV2373) from Novavax and COVOVAX (Novavax formulation) from Serum Institute of India) (3, 4). The strategy of all these vaccine platforms except inactivated virus vaccines is based on the SARS-CoV-2 spike protein. Among these vaccines, Pfizer/BioNTech was the first COVID-19 vaccine that has been approved by the U.S. Food and Drug Administration (FDA) in 2021. Subsequently, Moderna, Johnson & Johnson, and Novavax have also been approved by the FDA in 2022 (5, 6).

SARS-CoV-2 spike protein is a ~180 kDa glycoprotein that can form a trimeric structure that protrudes from the surface of the viral particle and play a key role in host cell entry (7–9). The total length of the SARS-CoV-2 spike protein contains 1273 amino acids (a.a) consisting of a signal peptide (a.a. 1–13 residues), S1 subunit (a.a. 14-685), and S2 subunit (a.a. 686-1273). The S1 subunit contains a N-terminal domain (a.a. 14–305), a receptor-binding domain (S1-RBD, a.a. 319-541), and a receptor-binding motif (RBM, a.a. 437–508). The S2 subunit includes the fusion peptide (a.a. 788–806), heptapeptide repeat sequence 1 (HR1, a.a. 912–984), heptapeptide repeat sequence 2 (HR2, a.a. 1163–1213), transmembrane domain (a.a. 1213–1237), and cytoplasm domain (a.a. 1237–1273) (10). The RBM is a portion of the S1-RBD that makes direct contact with the human cell surface receptor angiotensin-converting enzyme 2 (ACE2), whereas the S2 subunit mediates subsequent membrane fusion with the host cell membrane by forming a six-helical bundle *via* the two HR domain (7, 11). Within the S1 and S2 subunits, the S1-RBD has been considered an immunodominant region and the main target of neutralizing antibodies (12–14).

In Taiwan, four COVID-19 vaccines have been granted emergency use authorization (EUA) by Taiwan Food and Drug Administration (TFDA), including AZD1222 (Vaxzevria, Oxford/AstraZeneca), mRNA-1273 (Spikevax, Moderna), BNT162-b2 (Comirnaty, Pfizer/BioNTech), and MVC-COV1901 (Taiwan-based Medigen Vaccine Biologics Corporation) at the study time. With similar strategies to NVX-CoV2373 (Novavax), MVC-COV1901 is a protein subunit vaccine adjuvanted with CpG 1018 and aluminum hydroxide. To stabilize the prefusion form spike protein for preserving neutralizing epitopes, a GSAS replacement at the S1/S2 furin cleavage sites to confer protease resistance and two proline substitutions at residues 986 and 987 (K986P, V987P) in the sequence of the wild-type spike from the Wuhan strain were incorporated (15). Additionally, a trimerization domain (IZN4) is added to the C-terminus for improving the conformational homogeneity (16). According to the statistical data from the TFDA website (https://www.cdc.gov.tw/En/File/Get/BlkBAw7kMxwGx–DsPEvtg), a total of 63.17 million doses have been administered in Taiwan (mRNA-1273: 23.89 million doses, BNT162-b2: 19.3 million doses, AZD1222: 15.3 million doses, MVC-COV1901: 3.06 million doses) as of October 24^th^, 2022.

Vaccination reduces not only COVID-19 transmission, but also severe illness and deaths from COVID-19 infection (17). However, vaccines could not provide full protection from COVID-19 infection, especially when SARS-CoV-2 has an extremely high mutation rate (around 8×10^−4^ nucleotides/genome annually) compared to other viruses (18, 19). Indeed, the surveillance data showed that SARS-CoV-2 had generated a lot of mutations since 2019 (20, 21). According to the WHO classification, there were at least five variants of concerns (VOCs) that caused large outbreak waves in different countries in different periods, including B.1.1.7 (Alpha), B.1.351 (Beta), P.1 (Gamma), B.1.617.2 (Delta), and B.1.1.529 (Omicron). The possible attributes of VOCs include the evidence of higher transmissibility, increased virulence, and reduced effectiveness of vaccines, therapeutics, or diagnostics (22, 23). Currently, Omicron is the dominant variant circulating globally and accounting for nearly all sequences reported to GISAID (24). Omicron and its sublineages (BA.1, BA.2, BA.3, BA.4, BA.5, and descendent lineages) have significantly more mutations than previous SARS-CoV-2 variants particularly in the spike gene (25–27). Compared to the original strain, thirty amino acid changes (among which fifteen are in the RBD region), three deletions, and one insertion occur in the Omicron spike protein (27). Not only breakthrough infection, but the number of people reinfected with the coronavirus has increased since the Omicron variant spread globally (28–30).

As a part of the global value chain, Taiwan would surely loosen the broad restrictions (or lockdown restrictions). Therefore, evaluating the neutralizing activity especially against the SARS-CoV-2 variant of concerns is very important. Many factors affect immune responses to the SARS-CoV-2 vaccines, including age, gender, nutritional status, body mass index, host genetic polymorphism, chronic disease, and immune history (31, 32). More and more studies indicate that SARS-CoV-2 infection could cause immune disturbance and trigger autoantibodies production (33, 34). Several studies have shown that anti-ACE2 autoantibodies increase in COVID-19 patient serum and significantly positively correlate with disease severity (35, 36). However, whether COVID-19 vaccines would trigger anti-ACE2 autoantibodies or by which vaccine platform is unclear.

This study focused on the serological responses of various COVID-19 vaccines used in Taiwan by analyzing the production of anti-spike, anti-S1-RBD, and anti-ACE2 autoantibodies. In addition, the breadth of neutralizing antibody response was evaluated using a lentiviral pseudovirus system encoding the spike protein of ancestral SARS-CoV-2 and other six SARS-CoV-2 variants. Furthermore, the antigenic maps were generated by using the neutralizing titer and were rendered separately or combined with various vaccines.

## Materials and methods

### Cohort information

This study recruited Taiwanese who received two homologous doses of a COVID vaccine. The serum samples were collected four weeks after the second dose of vaccination. Four different COVID-19 vaccine brands were included, including AZD1222 (Vaxzevria, Oxford/AstraZeneca), mRNA-1273 (Spikevax, Moderna), BNT162-b2 (Comirnaty, Pfizer/BioNTech), and MVC-COV1901 (Taiwan-based Medigen Vaccine Biologics Corporation). The pre-pandemic serum samples from the healthy donors were collected before 2019 and used as the negative control sera.

### Serum collection and storage

All blood samples were processed on the collection day in a single-step standardized method. Briefly, whole blood was collected in red-topped vacutainers, plastic vacutainers containing clot activators but no anticoagulants (BD Biosciences, Franklin Lakes, New Jersey). The blood was allowed to clot by leaving it undisturbed at room temperature for 30 minutes. Sera were collected after centrifuging whole blood at 1500 ×g for 15 min at room temperature without brake. The undiluted sera were transferred and stored in polypropylene conical tubes at −80 °C for subsequent analysis. Before conducting the micro-neutralization assay, the serum was aliquoted and heat-inactivated at 56 °C for 30 min for complement inactivation.

### Recombinant proteins

C-terminal His-tag SARS-CoV-2 trimeric spike (extracellular domain, ECD) recombinant protein and N-terminal 6×His-SUMO tag SARS-CoV-2 nucleocapsid protein expressed from HEK293 were purchased from Leadgene Biomedical, Inc. Tainan, Taiwan. C-terminal 6×His and Avi-tagged human ACE2 (ECD) protein from HEK293 were purchased from GeneTex, Inc. Irvine, CA. C-terminal His-tagged SARS-CoV-2 S1-RBD recombinant proteins (YP_009724390) purified from S2 cells (37).

### Indirect enzyme-linked immunosorbent assay

An indirect ELISA was performed to quantify the levels of anti-nucleocapsid/spike/S1-RBD binding antibodies and anti-ACE2 autoantibodies in COVID post-vaccination sera. Briefly, the indicated proteins (2 µg/mL) were coated onto a high-binding 96-well ELISA plate overnight at 4°C. After blocking with 1% BSA in PBS, diluted sera (1:100 for anti-nucleocapsid/spike/S1-RBD binding antibodies, 1:50 for anti-ACE2 autoantibodies) were added and incubated in wells for 1 h at 37°C. The primary antibodies were allowed to bind to the anti-human IgG-HRP detection antibody (1:4000) (Thermo Fisher Scientific, Waltham, MA) for 1 h at 37°C. Wells were washed three times with PBST (PBS containing 0.01% Tween 20) between each step. For color visualization, the tetramethylbenzidine (TMB) reagent (Clinical Science Products, Mansfield, MA) was added to the wells for 10-15 min, and the reaction was stopped by the addition of 2N H_2_SO_4_. The absorbance at OD 450 nm was read by a VersaMax microplate reader (Molecular Devices, Sunnyvale, CA).

### Production of SARS-CoV-2 lenti-pseudovirus

We applied the lentiviral vector system provided by the National RNAi Core of Academia Sinica Taiwan to generate SARS-CoV-2 pseudovirus as mentioned previously (38). The sequences of SARS-CoV-2 full-length spike protein, including Wuhan (YP_009724390.1), B.1.1.7 (alpha), B.1.351 (beta), B.1.617.2 (delta), and B.1.1.529 (omicron) were optimized synthesized and cloned into a pcDNA3.1+ vector for expression (Leadgene, Tainan, Taiwan). The P. 1 (gamma) and C. 37 (lambda) were purchased from Sino Biological (Beijing, China). Several silent mutations were introduced into the full-length DNA sequence of SARS-CoV-2 Spike (S gene) to increase the protein expression level in mammalian cell system. HEK293T cells were co-transfected with pCMVdeltaR8.9, pLVX-NanoLuc-Puro (Leadgene, Tainan, Taiwan), and the plasmids expressing S gene for different SARS-CoV-2 variants were transfected into 293T cells using a TransIT-X2 transfection reagent (Mirus Bio LLC, Madison, Wisconsin). After 24 h transfection, the culture medium was displaced by FreeStyle™ 293 expression medium (Thermo Fisher Scientific, Waltham, MA) and then cultured for an additional 24 h. The lentiviral supernatant was further 30-fold concentrated using Lenti-X concentrator (Takara Bio, San Jose, CA), and the infectivity of SARS-CoV-2 lenti-pseudoviruses were determined by using the median tissue culture infectious dose (TCID_50_) in a HEK293-human ACE2 overexpression stable cell line (HEK293-ACE_O/E_) (Leadgene, Tainan, Taiwan).

### Commutability assessment

To convert the binding OD values and NT50 into binding antibodies units (BAU) and international units (IU), the COVID-19 patients standard sera calibrated by WHO international standard (IS) sera (20/130, 20/136, and 20/268) were kindly provided by Prof. Shin-Ru Shih (Chang Gung University). The 50% neutralization titer (NT50) values for standard patients sera were determined by pseudovirus micro-neutralization assay. To confirm the test accuracy of our pseudovirus micro-neutralization assay, another standard serum (COV 110-09) co-calibrated by five different labs in Taiwan, provided by Taiwan Food and Drug Administration (TFDA), was used. Each standard serum sample was tested in duplicate or triplicate independently.

### Pseudovirus micro-neutralization assay

The neutralizing antibody titer of each vaccinee against different SARS-CoV-2 variants of concern were tested using a lenti-pseudovirus system. HEK293-ACE_O/E_ cells were seeded on 96-well plates 18-24 h before infection. Complement-inactivated sera with the indicated dilution factors were preincubated with 100 TCID_50_ of SARS-CoV-2 spike-expressing pseudovirus for 1 h and added to the HEK293-ACE_O/E_ seeding plate. After 18-24 h incubation, the infection rate of SARS-CoV-2 lenti-pseudoviruses was evaluated using a Nano-Glo Luciferase Assay System (Promega, Madison, WI), and the luciferase signal was detected by a SpectraMax iD5 (Molecular Devices). The titers were determined using curve-fitting functions statistical packages (GraphPad Prism). The tabular neutralization data were analyzed manually and also using antigenic cartography.

### Antigenic cartography

Antigenic cartography is a method to visualize neutralization data by reflecting the antigenic properties of a pathogen. In an antigenic map, the positions and distances of antigens and sera represent their antigenic relationship. The difference between the log_2_ of the maximum titer observed and the log_2_ of the titer for the serum and antigen coincides with the distance between the serum and antigen. Thus, each titer in a neutralization assay can be thought of as specifying a target distance for the points in an antigenic map. In this study, the antigenic maps were generated using a web-based tool (Acmacs Web Cherry, an open resource available from https://acmacs-web.antigenic-cartography.org/).

### Statistical analysis

All data were analyzed by GraphPad Prism version 6.0 (GraphPad Software Inc., CA). The results were presented as geometric mean titers (GMT) with 95% confidence interval (CI). Student’s unpaired t-test was used to analyze the differences between two groups. One-way ANOVA with a Kruskal–Wallis comparison test was used to analyze the differences among multiple groups. The correlation between the neutralizing titers and the serological response against the proteins (trimeric spike, S1-RBD, and human ACE2) was calculated through Spearman’s correlation. P values <0.05 were considered statistically significant.

## Results

### Participants (Cohort information)

We enrolled 113 SARS-CoV-2-naïve participants and collected their sera samples within days 30 to 120 after the second dose of homologous COVID vaccination (**Figure S1A**) between December 2021-January 2022. To confirm the serological activity was only triggered by the vaccination but not by infection, the binding antibodies against SARS-CoV-2 nucleocapsid and trimeric spike proteins of all sera samples were tested. The pre-pandemic serum samples from the healthy donors were collected before 2019 and used as the negative control sera. As shown in **Figure S1B**, the mean optical densities (OD) of antibodies against nucleocapsid in the vaccinated sera has no significant difference from that in the pre-pandemic sera, suggesting all the participants had not been infected by COVID-19 before. The anti-SARS-CoV-2 spike binding antibody in the post-vaccination sera was significantly higher than in the pre-pandemic sera, indicating the robust antibodies response was triggered by the vaccination (**Figure S1C**).

To compare the serological response induced by different vaccine brands, the participants were further classified into four groups according to the received vaccine brands (including AZD1222 (n=42), mRNA-1273 (n=19), MVC-COV1901 (n=27), and BNT162-b2 (n=25)). Median age, gender ratio, average sample collection days after the second dose vaccination, and the detailed age/gender distribution of the participants in each group were shown in **Table 1** and **Figure S2**. The average sample collection days after the second dose vaccination of the MVC-COV1901 group (69.46 days) was significantly higher than the other three groups (42.1 days in the AZD1222 group, 46.74 days in the mRNA-1273 group, and 45.08 days in the BNT162-b2 group) (**Figure S1D**), while there was no significant difference in the mean ages between each group (**Figure S1E**).

**Table 1:** Demographic characteristics of vaccinated individuals.

Sample group	Case Number	Average Age	Gender	Average Collection time (days)
ChAdOx1 nCoV-19 (AZD1222, AZ)	42	35.26	M: 40.5% F: 59.5%	42.1
mRNA-1273 (Moderna)	19	33.37	M: 26.4% F: 73.6%	46.74
MVC-COV1901 (Medigen)	27	31.00	M: 51.8% F: 48.2%	69.46
BNT162b2 (Pfizer- BioNTech, BNT)	25	33.71	M: 36.0% F: 64.0%	45.08

28-120 days after homologous 2^nd^ vaccine dose.

### The serological response triggered by different vaccine brands

#### Anti-spike and anti-S1-RBD antibodies response

The serological response triggered by different vaccine brands was evaluated by the anti-spike and anti-S1-RBD binding antibodies quantification using ELISA, and the neutralizing (NT) antibody titers were determined by NT50 value in a lenti-pseudovirus neutralization assay. The binding OD values and NT50 values were converted into binding antibodies units (BAU) and international units (IU), respectively, using WHO standard calibrated sera that were kindly provided by Prof. Shin-Ru Shih (**Figure S3**).

**Figure 1:**
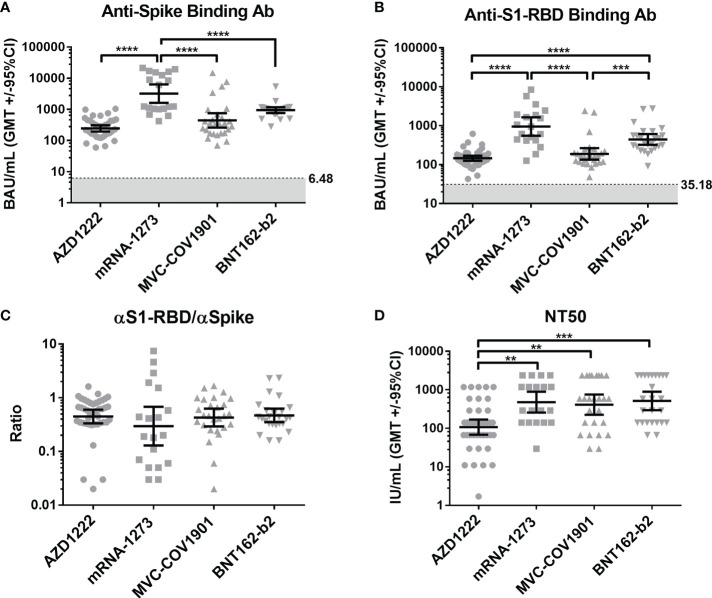
Compare the serological response triggered by different vaccine brands. **(A)** The levels of anti-spike binding antibody and **(B)** anti-S1-RBD binding antibody in the sera of four vaccinee groups (AZD1222, mRNA-1273, MVC-COV1901, and BNT162-b2) were evaluated by indirect ELISA. The mean value from the pre-pandemic serum group was determined as the cutoff value (grey). **(C)** The ratio of anti-S1-RBD to anti-spike binding antibody was shown. **(D)** Comparison of sera’s neutralizing antibodies titers (NT50) in different COVID-19 vaccine groups. **P < 0.01, ***P < 0.001, ****P < 0.0001,.

The geometric mean end-point titers (GMTs) of the anti-spike binding antibodies in the mRNA-1273 sera group were 3214 BAU/mL (95% confidence interval [CI], 1626 to 6353 BAU/mL), which were significantly higher than in AZD1222 (GMTs, 245.4 BAU/mL, 95% CI, 194.9 to 308.9 BAU/mL), MVC-COV1901 (GMTs, 442.8 BAU/mL, 95% CI, 226.3 to 753.3 BAU/mL), and BNT162-b2 (GMTs, 944.3 BAU/mL, 95% CI, 758.6 to 1176 BAU/mL) vaccinees (**Figure 1A**). Notably, we observed that mRNA-1273 recipients show a bimodal distribution in anti-spike binding antibodies (**Figure 1A** and **S4A**). However, there were no significant difference in the neutralizing activity (**Figure S4B**). We therefore separated the mRNA-1273 recipients into two groups: high- and low-anti-spike antibody production groups (**Figure S4A**), and further investigated the factors involved in the bimodal distribution (**Figure S4**). As shown in **Figure S4**, age might be responsible for the differences in anti-spike binding antibody production, but not sample collection time (**Figure S4C, 4D, 4E, and 4F**). The GMTs of the anti-S1-RBD binding antibodies in the mRNA-1273 and BNT162-b2 vaccinees were 950 BAU/mL (95% CI, 550.8 to 1639 BAU/mL) and 441.8 BAU/mL (95% CI, 321.2 to 607.6 BAU/mL), that were significantly higher than in AZD1222 (GMTs, 145.8 BAU/mL, 95% CI, 125.4 to 169.5 BAU/mL) and MVC-COV1901 (GMTs, 188.6 BAU/mL, 95% CI, 134 to 265.3 BAU/mL) vaccinees (**Figure 1B**). Since the S1-RBD has been considered an immunodominant region and the main target of neutralizing antibodies. We further compared the ratios of antibodies targeting S1-RBD to total anti-spike binding antibodies in the different vaccine sera groups. We found no significant difference in the ratios of anti-S1-RBD to anti-spike binding antibodies between each vaccine sera group (**Figure 1C**). In the pseudovirus neutralization test, the neutralizing activity of the AZD1222 antisera group (GMTs, 107.8 IU/mL, 95% CI, 68.52 to 169.7 IU/mL) was significantly lower than the other three groups (**Figure 1D**). There was no significant difference in the neutralizing activity between the mRNA-1273 (GMTs, 478.3 IU/mL, 95% CI, 255.3 to 896.1 IU/mL), BNT162-b2 (GMTs, 515.9 IU/mL, 95% CI, 297.3 to 895.3 IU/mL) and MVC-COV1901 (GMTs, 411.4 IU/mL, 95% CI, 224.5 to 754 IU/mL) vaccinees (**Figure 1D**). These results indicated that mRNA vaccines (mRNA-1273 and BNT162-b2) could elicit a more robust serological response, including anti-spike/S1-RBD binding and neutralizing antibodies, than adenovirus-based vaccine (AZD1222). In addition, the S-2P spike protein subunit vaccine, MVC-COV1901, was not inferior to mRNA vaccines in the ability to induce neutralizing antibodies production. ()

#### Anti-ACE2 antibodies triggered by SARS-CoV-2 vaccination

**Figure 2:**
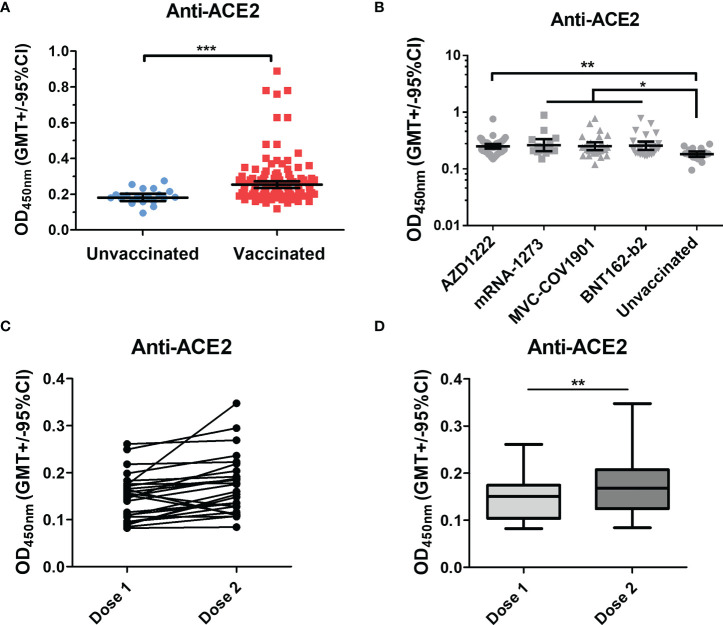
Anti-ACE2 Abs significantly increased in the COVID post-vaccination group. **(A)** Anti-ACE2 antibodies in the sera of pre-pandemic and vaccinated groups were tested. **(B)** The vaccinated group was separated into four groups (AZD1222, mRNA-1273, MVC-COV1901, and BNT162-b2), and the anti-ACE2 antibodies in the sera of these groups were compared with the pre-pandemic group. **(C, D)** Anti-ACE2 antibodies in the vaccinated sera after the first dose (dose 1) and the second dose (dose 2) were detected by an indirect ELISA. *P < 0.05, **P < 0.01, ***P < 0.001.

To compare the levels of anti-ACE2 antibodies in the sera of pre-pandemic and vaccinated subjects, we detected the binding antibodies (IgG) against human ACE2 using indirect ELISA. The mean OD of antibodies against human ACE2 in the sera of COVID-19 vaccinees was significantly higher than that in pre-pandemic cohorts (**Figure 2A**). In addition, four vaccinated groups showed a higher anti-ACE2 antibody level when compared with the pre-pandemic group (**Figure 2B**). However, there were no significant correlations between the levels of spike/S1-RBD binding antibodies and the levels of ACE2 antibodies in all vaccine types (**Figure S5**).

Moreover, we also compared the levels of anti-ACE2 antibodies in the sera of individuals after the first dose and the second dose of COVID-19 vaccination. As shown in **Figure 2C, D**, the levels of anti-ACE2 antibodies were significantly increased after the second dose of vaccination in the vaccinees. Overall, these results indicated that vaccination could induce the production of anti-ACE2 antibodies in individuals.

### Factors correlated to neutralizing titers

**Figure 3:**
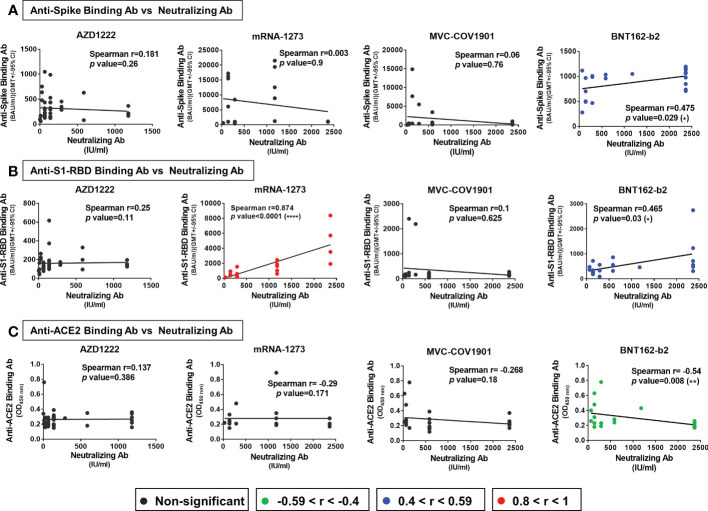
Factors correlated with neutralizing titers in different vaccine groups. The correlation of **(A)** anti-spike binding antibody levels **(B)** anti-S1-RBD antibody levels **(C)** anti-ACE2 antibody levels and neutralizing antibodies titers (NT50) of the AZD1222, mRNA-1273, MVC-COV1901, and BNT162-b2 vaccinees’ sera were evaluated. The levels of correlation coefficients are presented with different colors and the statistical significance in the correlations between each factor and neutralizing titers was shown. *P < 0.05, **P < 0.01, ****P < 0.0001.

Several studies have reported that anti-spike and anti-S1-RBD IgG levels in human serum/plasma positively correlate with neutralizing titer and represent the neutralization potency (39, 40). Here, we investigated the correlation between the neutralizing titers and the serological response against trimeric spike, S1-RBD, and ACE2 in different vaccinated groups. In the Spearman rank-order correlation results, anti-spike binding antibodies levels were not significantly correlated with NT titers in AZD1222 (r= 0.181; p=0.26), mRNA-1273 (r= 0.003; p=0.9), and MVC-COV1901 (r= 0.06; p=0.76) (**Figure 3A**). Moderate or weak correlation between neutralizing titers and anti-spike binding antibodies levels were observed in the BNT162-b2 vaccinee group (r= 0.475; p=0.029) and in total sera of post-vaccination groups (r= 0.307; p=0.001) (**Figures 3A**, **S6A**). On the other hand, anti-S1-RBD binding antibodies levels and NT titers were strongly correlated in mRNA-1273 vaccinee group (r= 0.874; p<0.0001), moderately correlated in BNT162-b2 (r= 0.465; p<0.03) and weakly correlated in total sera groups (r=0.373; p<0.03) (**Figure 3B** and **S6B**). However, no significant correlation between anti-S1-RBD binding antibodies levels and NT titers was observed in AZD1222 (*r*=0.221; p=0.1) and MVC-COV1901 (*r*=0.089; p=0.67) sera groups (**Figure 3B**). Interestingly, anti-ACE2 antibodies level was negatively correlated with the NT titers in the BNT162-b2 sera group (r=-0.54; p=0.008), but not in the other sera group (**Figure 3C** and **S6C**).

### Cross neutralizing activity to different SARS-CoV-2 variants

**Figure 4:**
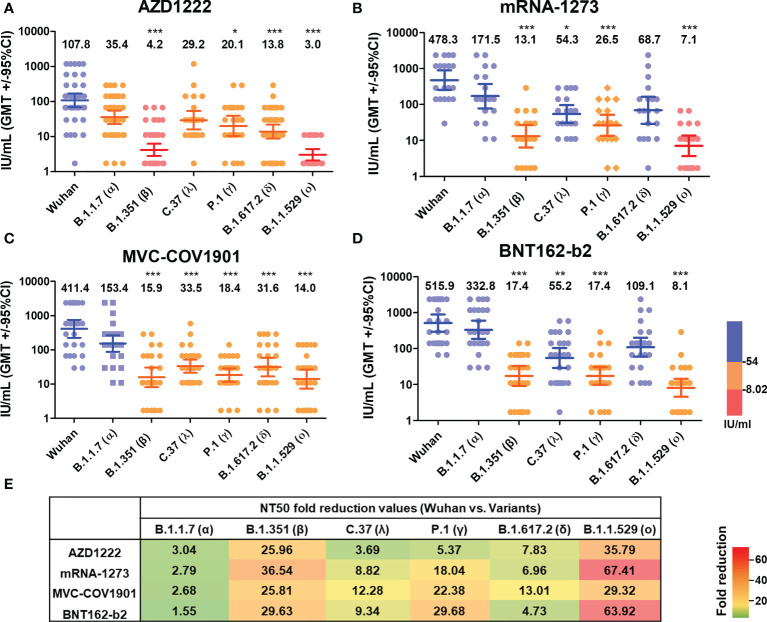
Neutralizing Ab response against different SARS-CoV-2 VOCs of different vaccine brands. **(A)** AZD1222, **(B)** mRNA-1273, **(C)** MVC-COV1901, and **(D)** BNT162-b2 vaccinees’ sera were tested by pseudovirus micro-neutralization assay. Each sample’s neutralizing antibodies titer (NT50) against different SARS-CoV-2 VOCs was shown. Low neutralizing antibody response was red, medium neutralizing antibody response was orange, and high neutralizing antibody response was blue. **(E)** Values of fold-reduction in the neutralization of Alpha, Beta, Lambda, Gamma, Delta, and Omicron are presented as heat maps with colors. The statistical difference compared to the Wuhan strain was shown. *P < 0.05, **P < 0.01, ***P < 0.001.

Next, we evaluated the neutralizing activity against different SARS-CoV-2 variants of different vaccinated groups using lenti-pseudovirus that encodes the spike protein of ancestral SARS-CoV-2 and other six SARS-CoV-2 variants (**Table 2**), including B.1.1.7 (alpha), B.1.351 (beta), C.37 (lambda), P.1 (gamma), B.1.617.2 (delta), and B.1.1.529 (omicron). According to a previous study, the antibody neutralization level of protection from symptomatic infection and protection from severe infection is 20.2% and 3.0% of the mean convalescent level, which correspond to approximately 54 IU/mL and 8.02 IU/mL, respectively (41). We, therefore, used the 54 and 8.02 IU/mL as the cut-off values to present the neutralizing levels. The neutralizing antibody mean titers higher than 54 IU/mL (blue), in the range of 8.02-54 IU/mL (orange), or lower than 8.02 IU/mL (red). In the AZD1222 sera group, the neutralizing activity exceeded 54 IU/mL was only for the ancestral Wuhan lineage (GMTs, 107.8 IU/mL, 95% CI, 68.52 to 169.7 IU/mL). The GMTs of NT50 against Alpha (35.44, 95% CI, 22.84 to 754.99), Lambda (29.18, 95% CI, 15.86 to 53.68), Gamma (20.08, 95% CI, 10.45 to 38.61), and Delta (13.77, 95% CI 8.68 to 21.87) were in the range of 8.02-54 IU/mL. The neutralizing titers against Beta (4.15, 95% CI 2.78 to 6.19) and Omicron (3.01, 95% CI 2.06 to 4.39) were decreased by a factor of 25.96 and 35.79, respectively (**Figure 4A, E**). In the MVC-COV1901 sera group, the GMTs of NT50 against ancestral lineage and Alpha reached 411.4 (95% CI 224.5 to 754) and 153.4 (95% CI 88.26 to 266.7). The GMTs of NT50 against Beta (15.94, 95% CI, 8.25 to 30.81), Lambda (33.49, 95% CI, 21.54 to 52.06), Gamma (18.38, 95% CI, 11.71 to 28.86), Delta (31.62, 95% CI 16.89 to 59.2) and Omicron (14.03, 95% CI 7.37 to 26.69) were in the range of 8.02-54 IU/mL (**Figure 4C**). As shown in **Figure 4B, D**, similar cross-neutralizing patterns with higher titers in the mRNA-1273 and BNT162-b2 vaccinee groups were observed. In detail, the GMTs of NT50 were 478.3 (95% CI 255.3 to 896.1), 171.5 (95% CI 78.18 to 376.2), 13.09 (95% CI 6.32 to 27.12), 54.25 (95% CI 30.66 to 95.99), 26.52 (95% CI 13.57 to 51.81), 68.7 (95% CI 28.95 to 163.0) and 7.09 (95% CI 3.68 to 13.69) for ancestral lineage, Alpha, Beta, Lambda, Gamma, Delta, and Omicron, respectively, in the mRNA-1273 vaccinee group (**Figure 4B**). The GMTs of NT50 were 515.9 (95% CI 297.3 to 895.3), 332.8 (95% CI 185.2 to 598.3), 17.41 (95% CI 9.26 to 32.72), 55.23 (95% CI 29.25 to 104.3), 17.38 (95% CI 9.84 to 30.71), 109.1 (95% CI 59.57 to 199.8), and 8.07 (95% CI 4.54 to 14.35) for ancestral Wuhan lineage, Alpha, Beta, Lambda, Gamma, Delta, and Omicron, respectively, in the BNT162-b2 vaccinee group (**Figure 4D**). Notably, none of the neutralizing titers against various variants was lower than 8.02 IU/mL in the MVC-COV1901, mRNA-1273, and BNT162-b2 sera group (**Figure 4C**). In addition, our results suggested that mRNA vaccines, including mRNA-1273 and BNT162-b2, elicit higher breadth of neutralizing antibody titers than MVC-COV1901, followed by AZD1222. The reduction neutralizing titers folds of each variant to the ancestral Wuhan were presented in **Figure 4E**. Compared with Wuhan, the reductions in neutralization against Omicron were the greatest in each vaccine group (about 65-fold in mRNA-1273 and BNT162-b2, 35.79-fold in AZD1222 and 29.32-fold in MVC-COV1901), followed by Beta (25.96-fold in AZD1222, 36.54-fold in mRNA-1273, 25.81-fold in MVC-COV1901 and 29.63-fold in BNT162-b2) and Gamma (5.37-fold in AZD1222, 18.04-fold in mRNA-1273, 22.38-fold in MVC-COV1901 and 29.63-fold in BNT162-b2). The reductions in neutralization against the other variants in each vaccine group were from 1.55-fold to 13.01-fold.

**Table 2:** Spike substitutions of all VOCs relative to ancestral Wuhan lineage in this study.

Variants of Concern	Spike mutation profile
B.1.1.7 (α)	69-70 del, 144Y del, N501Y, A570D, D614G, P681H, T716I, S982A, D1118H
B.1.351 (β)	L18F, D80A, D215G, 242-244 del, R246I, k417N, E484K, N501Y, D614G, A701V
C.37 (λ)	G75V, T76I, 246-252 del, D253N, L452Q, F490S, D614G, T859N
P.1 (γ)	L18F, T20N, P26S D138Y, R190S, K417N, E484K, N501Y, D614G, H655Y, T10271, V1176F
B.1.617.2(δ)	T19R, T95I, G142D, 156-157 del, R158G, L452R, T478K, D614G, P681R, D950N
B.1.1.529(o)	A67V, 69-70 del, T95I, 142-144 del, Y145D, 211 del, L212I, ins214EPE, G339D, S371L, S373P, S375F, K417N, N440K, G446S, 477N, T478K, E484A, Q493R, Q496S, Q498R, N501Y, Y505H, T547K, D614G, H655Y, N679K, P681H, N764K, D796Y, N856K, Q954H, N959K, L981F

### Antigenic cartography generated from homologous vaccinated sera with pseudovirus neutralizing assay

**Figure 5:**
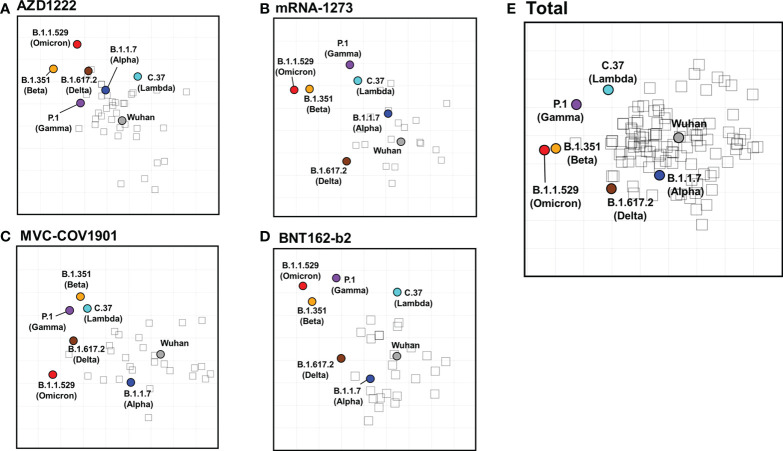
SARS-CoV-2 antigenic cartography generated from neutralizing antibodies response of different vaccine brands. Antigenic maps of SARS-CoV-2 VOCs were generated using neutralizing antibody titers from **(A)** AZD1222, **(B)** mRNA-1273, **(C)** MVC-COV1901, **(D)** BNT162-b2, and **(E)** total vaccinees’ sera. SARS-CoV-2 VOCs are shown as colored circles and sera are indicated as hollow squares. Both the x and y-axes of the map are antigenic distance, and each grid square (1 antigenic unit) represents a 2-fold change in neutralization titer.

Next, we constructed two-dimensional antigenic maps using neutralizing antibody titers from the sera of naïve vaccinees as described previously and were presented separately or combined with various vaccines (38, 42). Since the strategies of all four vaccine brands are based on the ancestral spike sequence, the sera tend to cluster around the ancestral Wuhan strain, reflecting that homologous neutralization is dominant. As shown in **Figure 5**, the maps generated from the neutralizing titers of mRNA-1273, MVC-COV1901, BNT162-b2, and total vaccinee groups were more similar than which generated from the neutralizing titers of AZD1222 group (**Figure 5A**). In the mRNA-1273 (**Figure 5B**), MVC-COV1901 (**Figure 5C**), BNT162-b2 (**Figure 5D**), and the total vaccinee maps (**Figure 5E**), the ancestral Wuhan and Alpha viruses cluster tightly together in the center of the map within 2 antigenic unit (1 unit = 2-fold change in neutralization titer). However, the distances between ancestral Wuhan and Lambda/Delta, Beta/Gamma, or Omicron were 2.48-3.83, 4.07-4.65, or more than 5 antigenic units, respectively (**Figure S7**). On the other hand, the lower neutralization titer to the ancestral Wuhan strain in AZ vaccinated group might result in a slight difference in the calculated distance between Wuhan to each variant. Indeed, all the antigenic distances between Wuhan to each variant below 4 in the map generated from the AZD1222 vaccine group (**Figure S7**).

## Discussion

In this study, we compared the serological response of naïve individuals with various homologous SARS-CoV-2 vaccine platforms. Consistent with the previous studies, mRNA vaccines (mRNA-1273 and BNT162-b2) could elicit more robust serological responses (anti-spike and anti-S1-RBD binding antibodies production and broadly neutralizing antibodies titer) than other platforms (AZD1222 and MVC-COV1901) (43–45). Notably, although the MVC-COV1901 vaccinee group had a lower amount of anti-spike and anti-S1-RBD binding antibodies than mRNA-1273 and BNT162-b2 groups, no significant difference in neutralizing antibody titers of them was found. In addition to vaccine platform, the strategies for SARS-CoV-2 spike protein sequence design might affect the vaccine efficacy (4, 46). There are several different strategies to stabilize the prefusion form spike protein. One is adding mutations at the S1/S2 furin cleavage sites to confer protease resistance; another is incorporating two proline substitutions at residues 986 and 987 (K986P, V987P) in the sequence of the wild-type spike from the Wuhan strain (47). Moreover, MVC-COV1901 adds a trimerization domain to the C-terminal for improving the conformational homogeneity (16). The mRNA-1273, BNT162-b2, and MVC-COV1901 use similar strategies to stabilize the prefusion form spike protein for preserving neutralizing epitopes; however, AZD1222 does not. This could be the possible reason for AZD1222 showing a lower neutralizing activity, compared with mRNA-1273, BNT162-b2, and MVC-COV1901 in this study.

Several limitations that should be taken into consideration in this study include the sample collection time period, sample sizes, and the use of a SARS-CoV-2 pseudovirus system for determining the neutralizing antibodies titers. In this study, only the MVC-COV1901 group had a significantly higher average sample collection days compared to the other groups. In addition, we found that there was no significant correlation between the neutralizing antibody titers and the time of sample collection (**Figure S8**). However, the influence of different sample collection times on neutralizing titers cannot be ignored. On the other hand, pseudoviruses can only replicate for a single round and the conformation/distribution of the spike protein on the pseudotyped virus may not exactly reflect the natural state of spike proteins on real SARS-CoV-2 (48). The neutralizing antibodies titers might be overestimated in the SARS-CoV-2 pseudovirus system. Indeed, inflating titers for the particular SARS-CoV-2 gamma (P.1) variant was observed in other studies using lentiviral pseudotype virus (49).

According to the statistical data from the TFDA website (https://www.cdc.gov.tw/En/File/Get/YTqTkmtAHH7fmKY6JF3l_A), 93.8% of the population has received at least one dose of a COVID-19 vaccine, 88.2% of the Taiwanese population have received two doses of the COVID-19 vaccines, and 73.8% of persons have received the booster dose as of October 24^th^, 2022. A total of 63.18 million doses have been administered in Taiwan (mRNA-1273: 23.89 million doses, BNT162-b2: 19.3 million doses, AZD1222: 15.3 million doses, MVC-COV1901: 3.06 million doses) and 20,172 cases of vaccine adverse reactions have been reported (AZD1222: 8,541; mRNA-1273: 5,513; MVC-COV1901: 826; BNT162-b2: 5,731). Although the proportion is very low, vaccine adverse reaction still causes concern to the public. One of the known causes of adverse vaccine reactions is autoimmune disease (50, 51). Indeed, many autoimmune phenomena, including vaccine-induced immune thrombotic thrombocytopenia, immune thrombocytopenic purpura, and rheumatoid arthritis, could be associated with COVID-19 vaccines (52–55). Several studies have reported that SARS-CoV-2 infection could induce the production of autoantibodies (33, 34, 56). Previous studies have shown that SARS-CoV-2 infection could induce the production of antibodies against ACE2 in the serum (35, 36, 57, 58), which might cause angiotensin II levels to increase and finally lead to a pro-inflammatory state (59). Thus, investigating whether COVID-19 vaccination induces the production of ACE2 auto-antibodies is needed. In this study, we compared the levels of ACE2 cross-reactive antibodies in the sera of individuals after the first dose and the second dose of vaccination. We found a significant increase in the levels of ACE2 cross-reactive antibodies in COVID-19 vaccinees. Some subjects showed an elevation in the levels of ACE2 cross-reactive antibodies after the second dose of vaccination. This indicated that the SARS-CoV-2 spike antigen induces the production of antibodies that can cross-react with ACE2.

With the increasing cases of re-infection and breakthrough, developing the next-generation SARS-CoV-2 vaccines is necessary. At this time, the public’s willingness to vaccinate seasonally is a big challenge. Since the durability of responses from different vaccine platform were comparable: the reduction folds at 180 days from the peak seen at 14 days after the second dose of neutralizing antibody titers were 4.5-7.1 in the mRNA platform (60, 61), 6.2 in the MVC-COV1901 (62), and 4.5 in AZD1222 (63). Increasing the breadth of neutralizing antibodies and reducing the vaccine adverse reaction will be the main goals for next-generation vaccines. In this study, we found that the mRNA vaccine platform could trigger robust serological response and the protein-subunit vaccine (MVC-COV1901) was able to induce neutralizing antibodies production as well as the mRNA vaccine. Therefore, we suggested that the design of spike with S-2P, furin-cleavage resistant and trimerization could be a mix strategy to the various vaccine platforms to produce the anti-spike/S1-RBD antibodies with neutralizing ability.

Another key point for the next-generation vaccine strategies would be to find a strain more suitable considering the new strains and outbreaks. Consistent with the antigenic maps have published by other groups, Omicron showed the farthest antigenic distance to Wuhan, implying Omicron represents very different antigenic characteristics from the ancestral lineage (64, 65). As autoimmune disease in COVID-19 has received attention, several studies have begun to examine whether SARS-CoV-2 vaccination can lead to autoantibodies production. However, the anti-ACE2 antibody was not commonly involved in the autoantibodies testing array (66). Here, we raised the issue that SARS-CoV-2 vaccines might trigger the anti-ACE2 antibodies production. Therefore, whether SARS-CoV-2 vaccines-induced anti-ACE2 autoantibody plays any biological function and the mechanisms of how SARS-CoV-2 vaccines lead to ACE2 autoantibodies production needs further investigation.

## Data availability statement

The datasets presented in this study can be found in online repositories. The names of the repository/repositories and accession number(s) can be found in the article/**Supplementary Material**.

## Ethics statement

The studies involving human participants were reviewed and approved by The Institutional Review Board of National Cheng Kung University Hospital (NCKUH) (IRB #A-BR-101–140, IRB #A-ER-110-423). The patients/participants provided their written informed consent to participate in this study.

## Author contributions

J-RW conceived the experiments. C-HC designed and performed the experiments and analyzed the data. C-HC and J-RW wrote and edited the paper. DC provided English editing and comments/suggestions for the manuscript. S-WH provided comments and suggestions during the preparation of the manuscript. Y-CC and T-MY shared the materials used in this study. All authors contributed to the article and approved the submitted version.

## Funding

This study was supported by the Ministry of Science and Technology of Taiwan (MOST 109-2327-B-006-005, MOST 111-2321-B-006-009); and National Health Research Institutes (NHRI IV-111-PP-06).

## Acknowledgments

We thank Prof. Shin-Ru Shih and Dr. Yu-An Kung (Research Center for Emerging Viral Infections, College of Medicine, Chang Gung University, Taoyuan City, Taiwan) provided the sera calibrated by WHO standard sera for the commutability assessment. We thank TFDA who provided the sera calibrated by five independent labs in Taiwan. We also thank the study participants who donated specimens for this study.

## Conflict of interest

Author Y-CC is employed by Leadgene Biomedical, Inc.

The remaining authors declare that the research was conducted in the absence of any commercial or financial relationship that could be considered a potential conflict of interest.

## Publisher’s note

All claims expressed in this article are solely those of the authors and do not necessarily represent those of their affiliated organizations, or those of the publisher, the editors and the reviewers. Any product that may be evaluated in this article, or claim that may be made by its manufacturer, is not guaranteed or endorsed by the publisher.
