# Supplementary material for: Serological responses triggered by different SARS-CoV-2 vaccines against SARS-CoV-2 variants in Taiwan
Source: Front Immunol. 2022 Nov 15;13:1023943. doi: 10.3389/fimmu.2022.1023943 (PMC9705976; doi:10.3389/fimmu.2022.1023943)
Supplement: Supplementary file 1 [file DataSheet_1.docx]

**SUPPLEMENT INFORMATION**

**
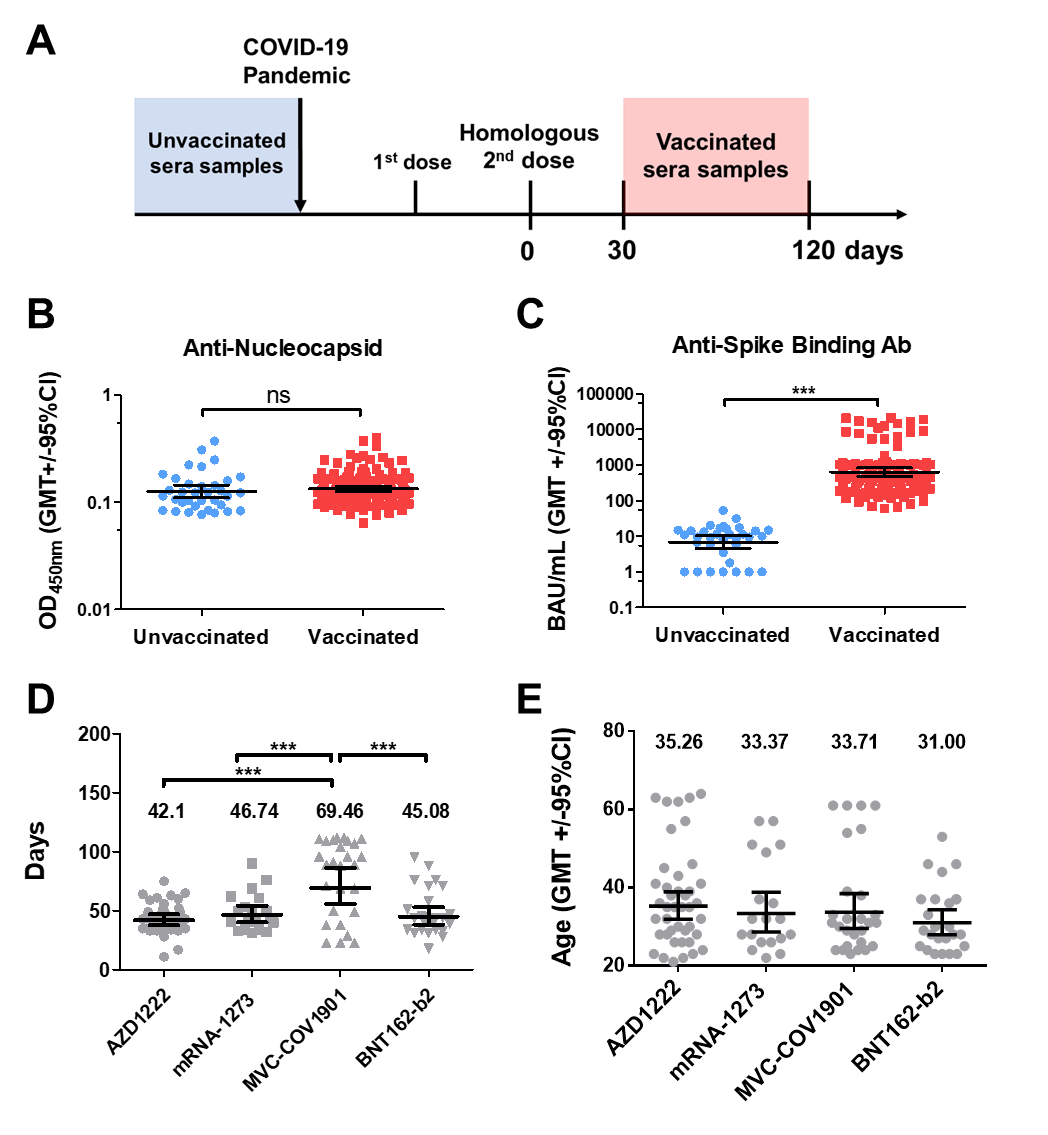
**

**Fig S1. Cohort information in this study. (A)** The collection period of the serum samples. **(B)** The anti-nucleocapsid antibodies and **(C)** the anti-spike antibody in the sera of both pre-pandemic and COVID-19 vaccinated groups were tested. The vaccinated group was separated into four groups (AZD1222, mRNA-1273, MVC-COV1901, and BNT162-b2). **(D)** The average serum sample collection days and **(E)** the average donors’ age of each group were shown. ***P < 0.001

**
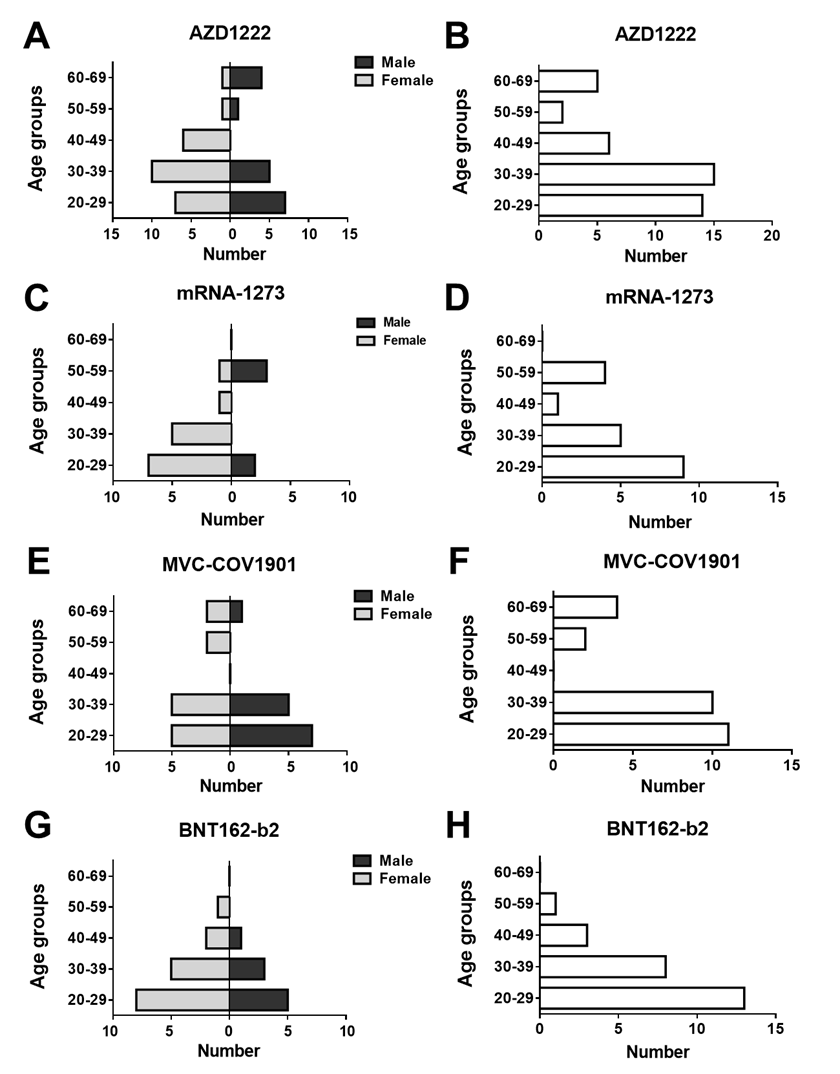
**

**Fig S2. Sex and age distribution.** The bar chart shows the age distribution of all vaccinated sera samples, combined with the gender (left) or not (right). The vaccinated sera samples, including **(A) (B)** AZD1222 vaccinated group, **(C) (D)** mRNA-1273 vaccinated group, **(E) (F)** MVC-COV1901 vaccinated group, and **(G) (H)** BNT162-b2 vaccinated group, were analyzed.

**
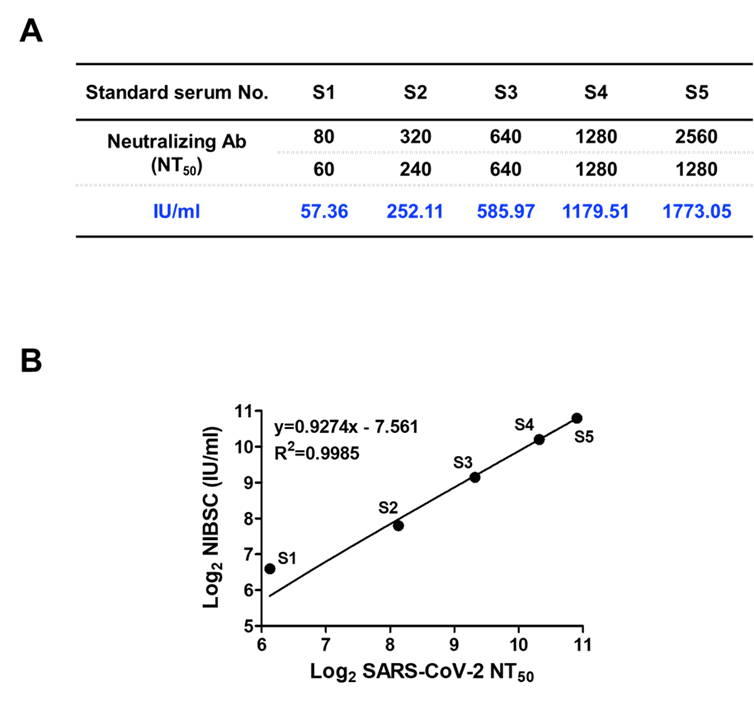
**

**Fig S3. Commutability assessment. (A)** Five sera samples calibrated by WHO standard sera (blue) were used to evaluate the NT50 value (black, two independent experiments) tested by the lenti-pseudovirus system in our study. **(B)** The standard calibration curve with the data S1 to S5 (black points) we tested using pseudovirus micro-neutralization assay was shown with an R^2^ value**.**


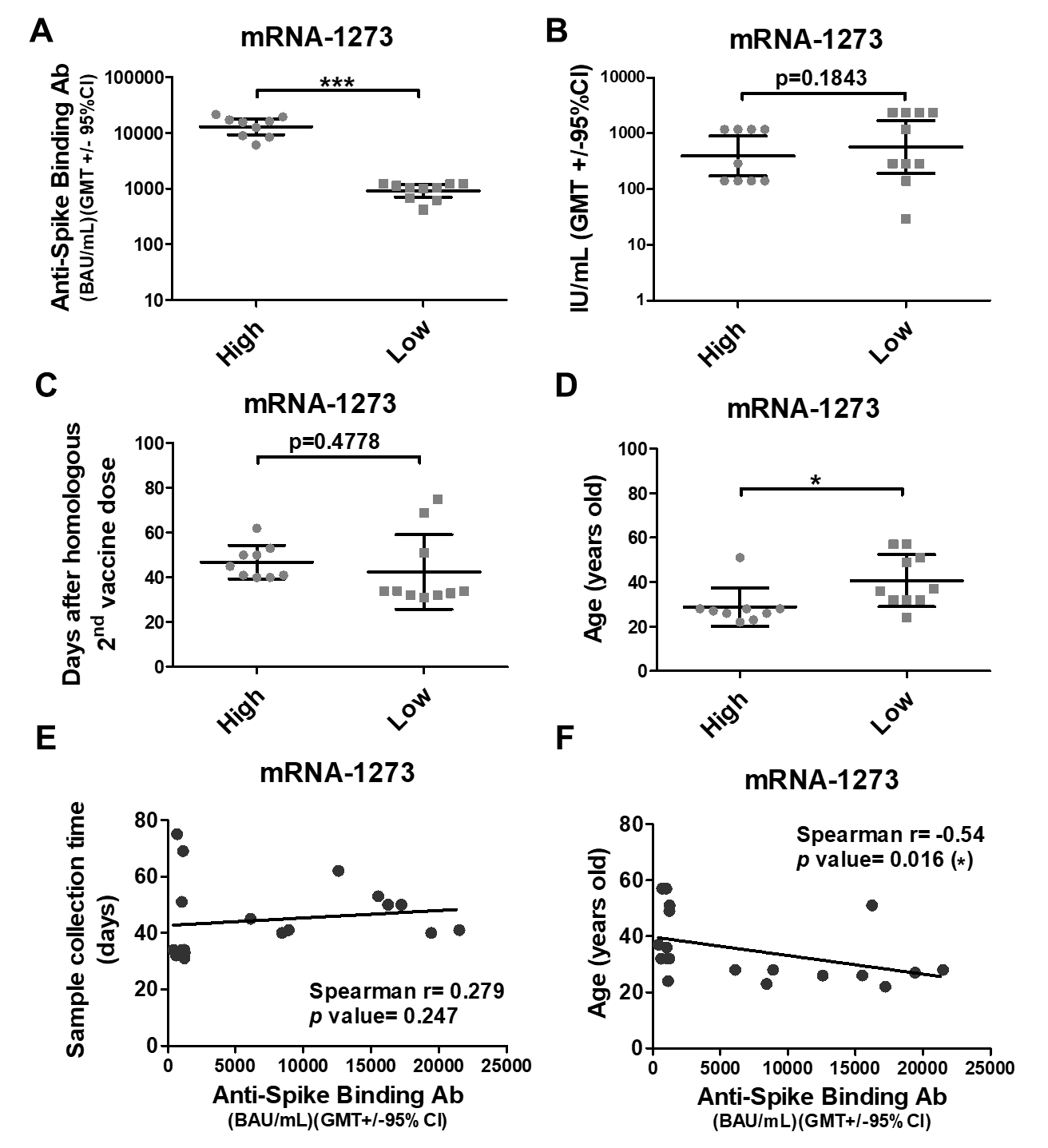


**Fig S4. Age, but not sample collection time, might be responsible for the differences in anti-spike binding antibody production in** **the mRNA-1273 vaccine group. (A)** The levels of anti-spike binding antibody **(B)** the neutralizing antibodies titers **(C)** the sample collection time (days) and **(D)** the mean age in mRNA-1273 vaccine group were presented. The correlations between anti-spike antibody levels and **(E)** sample collection time (days) or **(F)** the age were evaluated. *P < 0.05


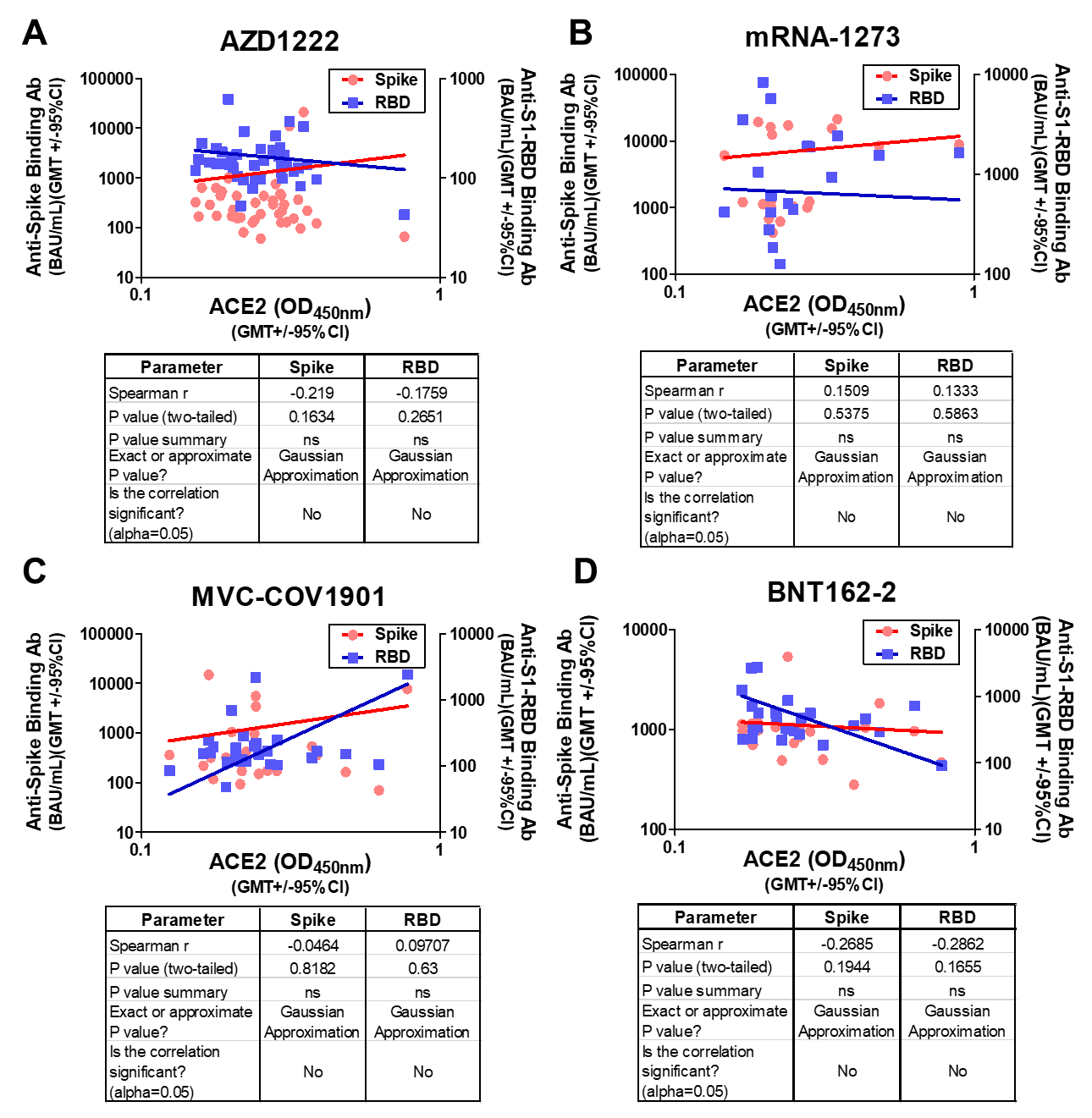


**Fig S5. The correlation between** **the levels of spike/S1-RBD antibodies and the levels of ACE-2 antibodies in (A) AZD1222 (B) mRNA-1273 (C) MVC-COV1901 and (D) BNT162-2.**

**
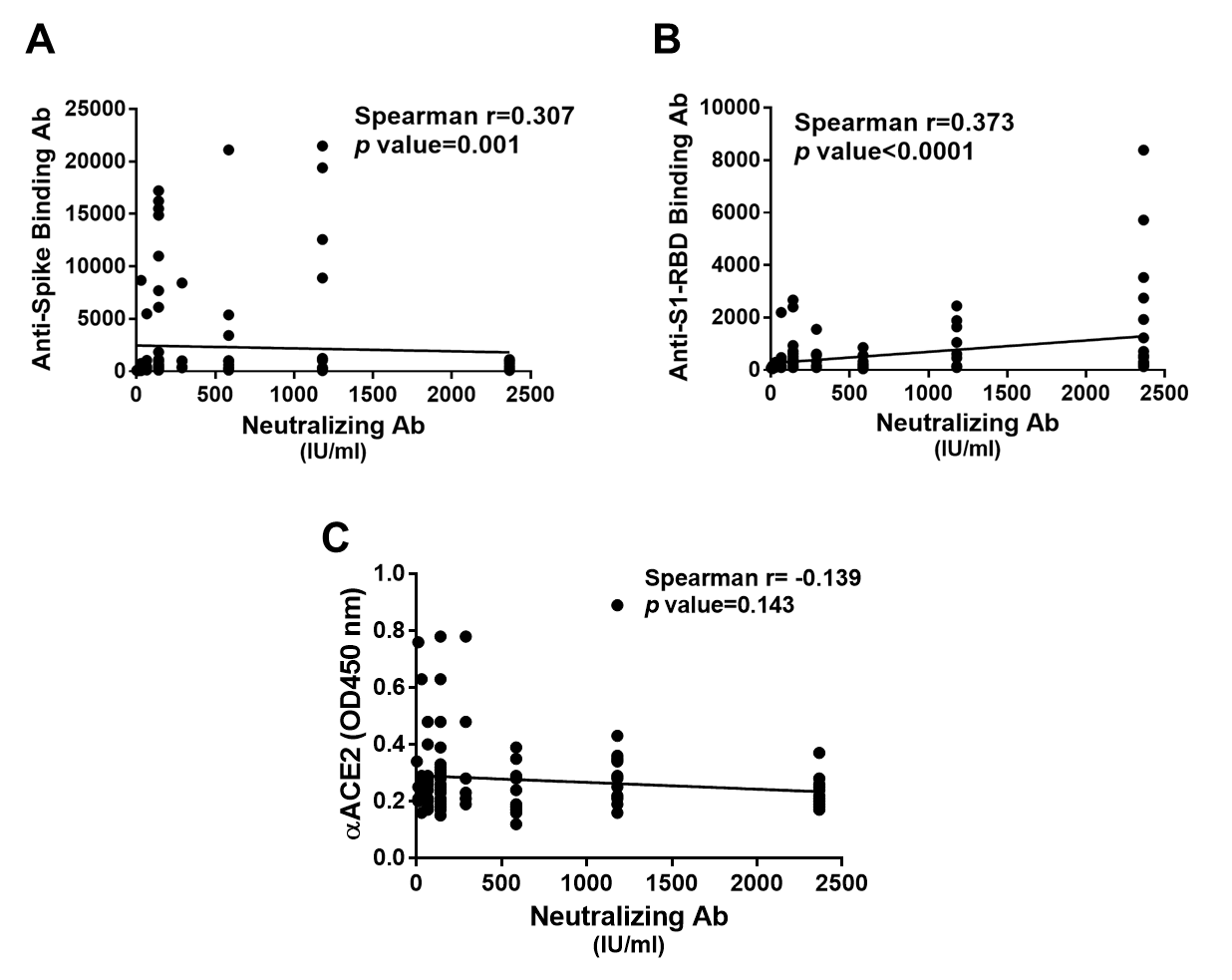
**

**Fig S6. Anti-S1-RBD binding Abs were more correlated with neutralizing titers in general. (A)** The correlations between anti-spike antibody levels and neutralizing antibodies titers (NT50) of all post-vaccination sera were evaluated. **(B)** The correlation between anti-S1-RBD antibody and NT50 or **(C)** the correlation between anti-ACE2 antibody and NT50 of all post-vaccination sera were also shown.

**
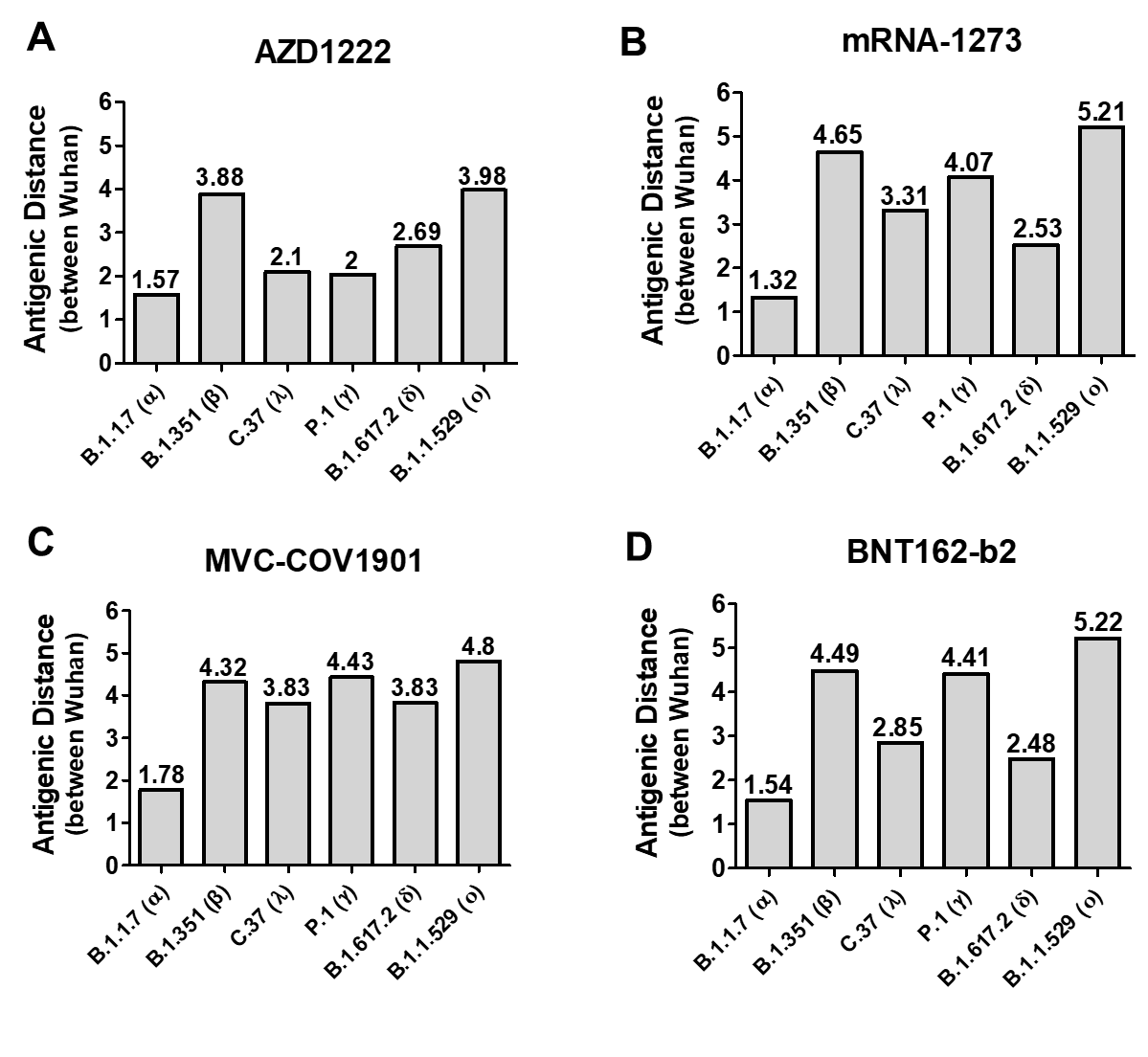
Fig S7. The antigenic distance between Wuhan to each variant.** The distance between ancestral Wuhan to different VOCs in the antigenic map generated from **(A)** AZD1222, **(B)** mRNA-1273, **(C)** MVC-COV1901, and **(D)** BNT162-b2 vaccinee’ antisera was calculated by Acmacs Web Cherry (an open resource available from https://acmacs-web.antigenic-cartography.org/).


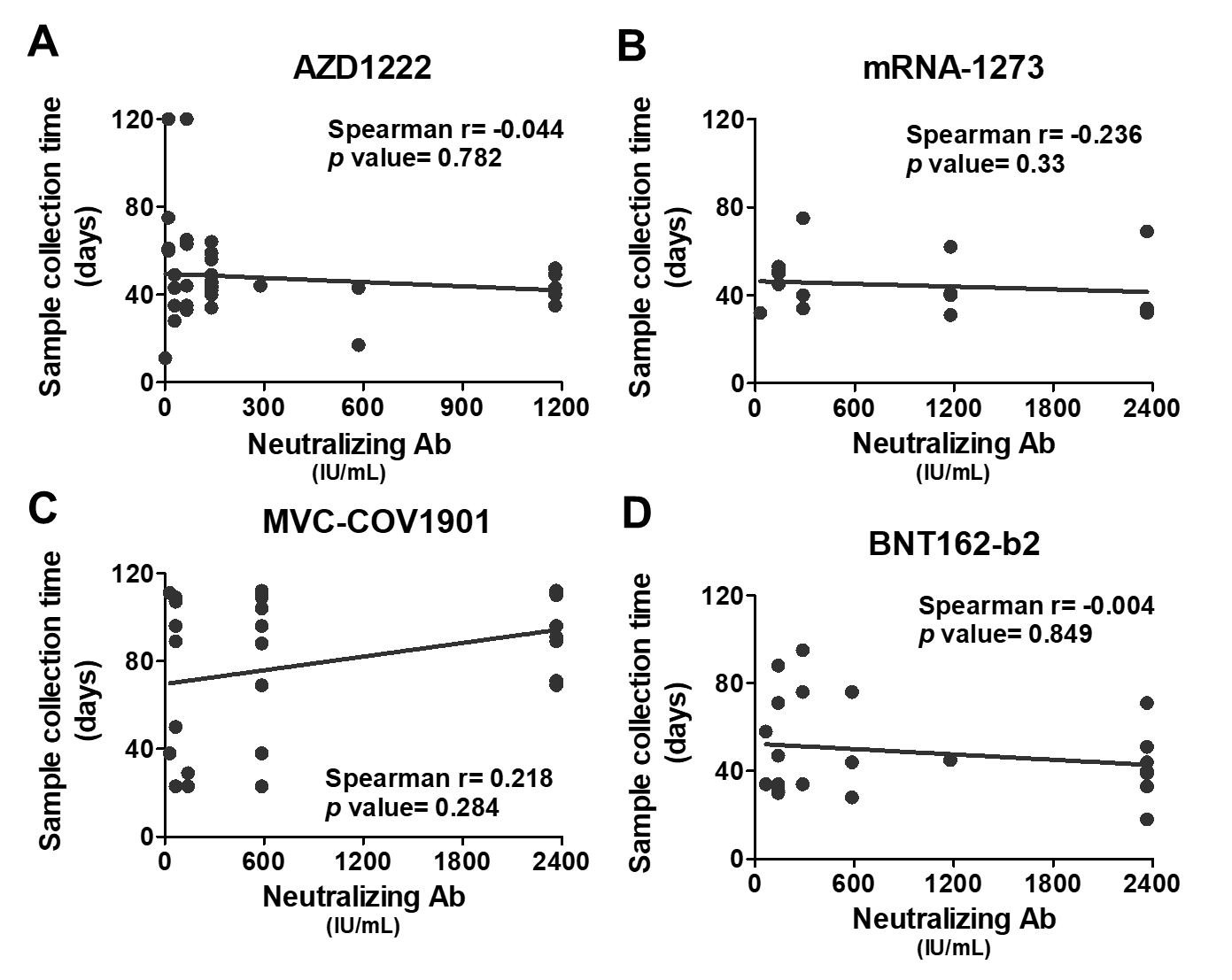


**Fig S8. No significant correlation between time of sample collection and neutralizing antibody activity within different vaccine types.** The correlations between neutralizing antibodies titers (NT50) and sample collection time (days) in **(A)** AZD1222 **(B)** mRNA-1273 **(C)** MVC-COV1901 and **(D)** BNT162-b2.
